# Editorial: Pharmacological mechanisms of drugs affecting bone formation and bone resorption

**DOI:** 10.3389/fphar.2023.1170340

**Published:** 2023-06-27

**Authors:** Dongwei Zhang, Xiaofeng Zhu, Alex Zhong

**Affiliations:** ^1^ Diabetes Research Center, School of Traditional Chinese Medicine, Beijing University of Chinese Medicine, Beijing, China; ^2^ Department of Chinese Medicine, The First Affiliated Hospital of Jinan University, Guangzhou, Guangdong, China; ^3^ Van Andel Institute, Grand Rapids, MI, United States

**Keywords:** bone metabolic diseases, bone remodeling, Chinese medicine, diabetic bone, energy metabolism

## 1 Introduction

Metabolic bone diseases are becoming a major health challenge in the aging population considering their high mortality and heavy economic burden on society (Shen et al., 2022). Ideally, osteoclastic resorption strives to keep pace with osteoblastic bone formation to maintain bone homeostasis (Chen et al., 2021). Disruption of the delicate balance between osteoblasts and osteoclasts leads to various bone diseases, such as osteoporosis, osteomalacia, osteogenesis imperfecta, osteopetrosis, and Paget’s disease of bone (Kim et al., 2020). Scientists and clinicians are actively exploring the underlying pathological mechanisms of bone disorders and seeking novel effective countermeasures to promote healthy bone remodeling (Srivastava et al., 2022; Xia et al., 2022).

### 1.1 Drugs, natural products, and pathology for bone remodeling

In this special collection, Lu et al. comprehensively examined the applications and limitations of the marketed anti-osteoporosis agents, highlighting the directions of the future drug candidates for the management of bone disorders. Li et al. and Wang et al. critically reviewed the recent advances in polyphenols and tanshinol-derived from traditional Chinese medicine (TCM) in maintaining the balance of bone formation and resorption. However, strong evidence from multicenter randomized trials is required to lay the foundation for clinical applications. Li et al. reviewed the role of autophagy in the development of metabolic bone diseases and examined the actions and challenges of natural products in the treatment of these diseases by targeting autophagy. Given that bone mineral density (BMD) examination may fail to provide a comprehensive profile of bone remodeling, Zeng et al. discovered that two differentially expressed genes—*METTL4* and *RAB2A*—are associated with BMD alterations, which contribute to accurate evaluation of the severity of osteoporosis and prediction of the risk of fracture.

### 1.2 Novel countermeasures to restore bone remodeling

New countermeasures, including synthetic compounds and TCM drugs, are emerging to restore bone remodeling. In this Research Topic, Wang et al. reported that N-[2-bromo-4-(phenylsulfonyl)-3-thienyl]-2-chlorobenzamide (BNTA), an artificially synthesized compound, can inhibit osteoclast formation and osteolytic resorption by suppressing the overaccumulation of intracellular reactive oxygen species (ROS) and receptor activator of nuclear factor kappa B ligand (RANKL)-stimulated proinflammatory cytokines to attenuate MARK signaling. In addition, Wu et al. reported that CDZ173, a selective PI3K inhibitor, might inhibit lipopolysaccharide-induced osteolysis by weakening the signal axis of PI3K-AKT/MAPK-NFATc1 in osteoclasts. Regarding the role of Chinese medicine in the regulation of bone homeostasis, several formulas and drugs have been extensively investigated, including Bu-Gu-Sheng-Sui decoction (Liu et al.), Yi Shen Juan Bi Pill (Xu et al.), QiangGuYin (Yuan et al.), monascin (Cheng et al.), and Shujin Huoxue Tablet (Sin et al.). The mechanisms of action of these TCM therapies in the promotion of healthy bone remodeling may be associated with the regulation of ERK/Smad, Ephrin B2, Wnt/β-catenin, JNK, and MAPK signaling cascades. Furthermore, we discuss the potential of monascin in preventing bone loss by inhibiting osteoclast activation. Interestingly, red yeast rice (RYR), one of the main sources of monascin, has been reported to maintain bone health (Wu et al., 2020), which highlights the potential of functional food for the management of chronic bone loss.

With the increasing prevalence of diabetes, the incidence of diabetic osteoporosis is also increasing every year (Ma et al., 2016). Currently, few drugs are available for managing this condition, sometimes termed “sweet and brittle bone disease.” (Ala et al., 2020). In this special collection, Chen et al. reported that ginsenoside Rg1 prevents the development of diabetic osteoporosis through the regulation of angiogenesis and osteogenesis coupling in Goto–Kakizaki rats. Emerging evidence suggests that ginseng attenuates the development of diabetic microvascular diseases (Liu et al., 2021; Zhang et al., 2022).

### 1.3 Perspectives

In addition, it is recognized that bone remodeling is highly integrated with energy metabolism, which is in turn regulated by endocrine factors. This may highlight that the musculoskeletal system functions are highly influenced by interactions among the adipose tissues, muscles, and bones (Gomes et al., 2022). Therefore, further investigations are required to study the pharmacological effects of anti-osteoporotic drugs on energy metabolism.

In summary, phytochemicals with therapeutic and preventive effects on bone metabolism play a significant role in the prevention of bone disorders, including osteoporosis. Elucidating the pathological mechanisms and discovering more reliable diagnostic markers of osteoporosis continue to be research frontiers.

## References

[B1] Ala M. Jafari R. M. Dehpour A. R. (2020). Diabetes mellitus and osteoporosis correlation: Challenges and hopes. Curr. Diabetes Rev. 16, 984–1001. 10.2174/1573399816666200324152517 32208120

[B2] Chen B. Wei J. Zhu R. Zhang H. Xia B. Liu Y. (2021). Fructus Ligustri Lucidi aqueous extract promotes calcium balance and short-chain fatty acids production in ovariectomized rats. J. Ethnopharmacol. 279, 114348. 10.1016/j.jep.2021.114348 34153448

[B3] Gomes M. M. da Silva M. M. R. de Araujo I. M. de Paula F. J. A. (2022). Bone, fat, and muscle interactions in health and disease. Arch. Endocrinol. Metab. 66, 611–620. 10.20945/2359-3997000000550 36382750 PMC10118823

[B4] Kim J. M. Lin C. Stavre Z. Greenblatt M. B. Shim J. H. (2020). Osteoblast-osteoclast communication and bone homeostasis. Cells 9, 2073. 10.3390/cells9092073 32927921 PMC7564526

[B5] Liu Y. Zhang H. Dai X. Zhu R. Chen B. Xia B. (2021). A comprehensive review on the phytochemistry, pharmacokinetics, and antidiabetic effect of Ginseng. Phytomedicine 92, 153717. 10.1016/j.phymed.2021.153717 34583224

[B6] Ma R. Zhu R. Wang L. Guo Y. Liu C. Liu H. (2016). Diabetic osteoporosis: A review of its traditional Chinese medicinal use and clinical and preclinical research. Evid. Based Complement. Altern. Med. 2016, 3218313. 10.1155/2016/3218313 PMC5028800 27698674

[B7] Shen Y. Huang X. Wu J. Lin X. Zhou X. Zhu Z. (2022). The global burden of osteoporosis, low bone mass, and its related fracture in 204 countries and territories, 1990-2019. Front. Endocrinol. (Lausanne) 13, 882241. 10.3389/fendo.2022.882241 35669691 PMC9165055

[B8] Srivastava R. K. Sapra L. Mishra P. K. (2022). Osteometabolism: Metabolic alterations in bone pathologies. Cells 11, 3943. 10.3390/cells11233943 36497201 PMC9735555

[B9] Wu B. Huang J. F. He B. J. Huang C. W. Lu J. H. (2020). Promotion of Bone Formation by red yeast rice in experimental animals: A systematic review and meta-analysis. Biomed. Res. Int. 2020, 7231827. 10.1155/2020/7231827 32832555 PMC7429765

[B10] Xia B. Zhu R. Zhang H. Chen B. Liu Y. Dai X. (2022). Lycopene improves bone quality and regulates AGE/RAGE/NF-кB signaling pathway in high-fat diet-induced obese mice. Oxid. Med. Cell. Longev. 2022, 3697067. 10.1155/2022/3697067 PMC8872668 35222796

[B11] Zhang H. Hu C. Xue J. Jin D. Tian L. Zhao D. (2022). Ginseng in vascular dysfunction: A review of therapeutic potentials and molecular mechanisms. Phytother. Res. 36, 857–872. 10.1002/ptr.7369 35026867

